# Unexpected structural complexity of supernumerary marker chromosomes characterized by microarray comparative genomic hybridization

**DOI:** 10.1186/1755-8166-1-7

**Published:** 2008-04-21

**Authors:** Karen D Tsuchiya, Kent E Opheim, Mark C Hannibal, Anne V Hing, Ian A Glass, Michael L Raff, Thomas Norwood, Beth A Torchia

**Affiliations:** 1 Department of Laboratories, Children's Hospital & Regional Medical Center, Seattle, WA, USA; 2 Department of Laboratory Medicine, University of Washington School of Medicine, Seattle, WA, USA; 3 Department of Pediatrics, University of Washington School of Medicine, Seattle, WA, USA; 4 Department of Pathology, University of Washington School of Medicine, Seattle, WA, USA; 5 Signature Genomic Laboratories, LLC, Spokane, WA, USA

## Abstract

**Background:**

Supernumerary marker chromosomes (SMCs) are structurally abnormal extra chromosomes that cannot be unambiguously identified by conventional banding techniques. In the past, SMCs have been characterized using a variety of different molecular cytogenetic techniques. Although these techniques can sometimes identify the chromosome of origin of SMCs, they are cumbersome to perform and are not available in many clinical cytogenetic laboratories. Furthermore, they cannot precisely determine the region or breakpoints of the chromosome(s) involved. In this study, we describe four patients who possess one or more SMCs (a total of eight SMCs in all four patients) that were characterized by microarray comparative genomic hybridization (array CGH).

**Results:**

In at least one SMC from all four patients, array CGH uncovered unexpected complexity, in the form of complex rearrangements, that could have gone undetected using other molecular cytogenetic techniques. Although array CGH accurately defined the chromosome content of all but two minute SMCs, fluorescence *in situ *hybridization was necessary to determine the structure of the markers.

**Conclusion:**

The increasing use of array CGH in clinical cytogenetic laboratories will provide an efficient method for more comprehensive characterization of SMCs. Improved SMC characterization, facilitated by array CGH, will allow for more accurate SMC/phenotype correlation.

## Background

Supernumerary marker chromosomes (SMCs) are structurally abnormal extra chromosomes that cannot be unambiguously identified by conventional banding techniques. A comprehensive description of SMCs can provide valuable information for genetic counseling, yet their extreme heterogeneity in size, structure, and chromosomal origin has resulted in limited characterization of many markers in clinical laboratories. In the case of SMCs containing satellites, targeted fluorescent *in situ *hybridization (FISH) testing for the most likely candidates, such as the inv dup(15q) and the inv dup(22q), are often employed; however, in the absence of an initial positive result, subsequent FISH analysis using individual probes becomes inefficient and costly. Furthermore, SMCs that lack satellites may have gone unclassified in many laboratories in the past.

A variety of molecular cytogenetic techniques that provide more comprehensive analysis in a single or a few experiments have been described for SMC characterization. Twenty-four color FISH, multicolor banding, centromere-specific multicolor FISH (cenM-FISH), subcentromere-specific multicolor FISH (subcenM-FISH), and microdissection followed by reverse FISH may all provide identification of the chromosome of origin of SMCs [1-4], but many of these techniques are not utilized by or accessible to most clinical laboratories. Even if 24-color FISH is readily available, this technique can result in ambiguous classification or misclassification of SMCs, particularly if they are small. In addition, these multicolor FISH techniques cannot precisely determine the chromosome regions or breakpoints involved.

Microarray-based comparative genomic hybridization (array CGH) is an efficient and sensitive technique for detecting genome-wide copy number alterations at high resolution [5]. We describe four patients who possess one or more SMCs (a total of eight SMCs in all four patients) that were characterized using a combination of G-banded analysis, FISH, and array CGH. Array CGH not only identified the chromosome of origin of all but two minute SMCs, but it also defined the region(s) and breakpoints of each chromosome involved. In addition, array CGH revealed unexpected structural SMC complexity that could easily have been missed if FISH and G-banding alone had been used for characterization.

## Results (summarized in Table 1)

**Table 1:** Summary of supernumerary marker chromosomes

Patient 1	Karyotype	46,XY (lymphocytes) 47,XY,+mar [8]/46,XY [29] (fibroblasts)
	Array result	arr cgh 3q22.3q29(RP11-306L14→RP11-159K3)x2~3
	FISH result	ish der(3)(q22.3qter)(wcp3+,D3Z1-,RP11-184L10-,RP11-976K13++,RP11-702G16++,qter++)
	SMC configuration	inv dup(3) (qter→q26→neo→q26→q22.3::q22.3→qter)
Patient 2	Karyotype	48,XY,+mar1,+mar2
	Array result	arr cgh 13q12.11(RP11-301J16,RP11-408E5,RP11-385E5)x3,13q33.3q34(RP11-54H7→RP11-569D9)x3
	FISH result	ish der(13)(p12qter)(NOR+,D13Z1/D21Z1+,RP11-347L8+,RP11-408E5+,RP11-63L17+)
	SMC configuration	13qter→q33.3::p12→q12.12:
Patient 3	Karyotype	47,XX,+mar
	Array result	arr cgh 22q11.1q11.21(RP11-701M12→CTD-2593O4)x3,22q13.31q13.33(RP11-281J5→GS1-99K24)x3
	FISH result	ish der(22)(q11.1qter)(RP11-1037C4+,CTD-2593O4+,RP11-676E13+)
	SMC configuration	22pter→q11.21::q13.31→qter
Patient 4	Karyotype	50,XX,+mar1,+mar2,+mar3,+mar4
	Array result	arr cgh 1p12(RP11-828N6→RP4-794L19)x3,4q12(RP11-39D6→RP11-231C18)x3,7p11.1(RP11-1324A7)x3,11q11q12.1(RP11-176J24→RP11-624G17)x3
	FISH result	ish der(X)(p11.1q11.1)(DXZ1+),der(1)(p12)(RP11-527D19+),der(7)(p11.1)(RP11-1324A7+),der(11)r(4;11)(::11q11→11q12.1::4q12::) (D4Z1-,RP11-601I15+,wcp11+,D11Z1+)
	SMC configuration	mar1 = der(11)r(4;11)(::11q11→11q12.1::4q12::) mar2 = der(7)(:p11.1:) mar3 = der(1)(:p12:) mar4 = der(X)(:p11.1q11.1:)

### Patient 1

**Figure 1:**
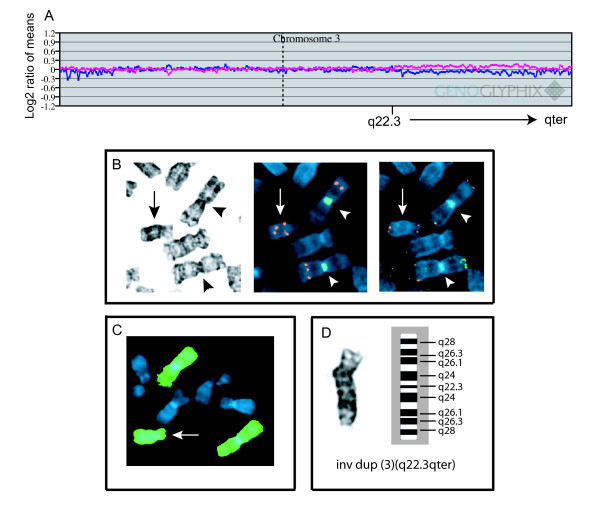
**Characterization of inv dup(3q) by array CGH and FISH**. A. Chromosome 3 array CGH plot. The X axis represents distal p arm to distal q arm (left to right), with the centromere designated by the vertical dotted line. The blue line is a plot of the results from an experiment of Cy5 labeled reference/Cy3 labeled patient, while the pink line is from a dye-reversal experiment (Cy5 patient/Cy3 reference). Slight separation of the plots from q22.3 to qter indicates low-level gain of this region due to marker mosaicism. B. Marker chromosome (arrow) and normal chromosome 3 homologues (arrowheads) from the same metaphase cell that was initially G-banded (left panel), then destained for FISH analyses (middle and right panels). The middle panel shows results of FISH using BAC RP11-976K13 from 3q25.32 (orange signal) and a chromosome 3 alpha satellite probe (green). Alpha satellite signal is present on the two normal homologues of chromosome 3, but absent from the inv dup(3q). The inv dup(3q) shows two sets of signals from BAC RP11-976K13. The right panel shows results of rehybridization using a subtelomeric probe mixture for chromosome 3 (red signal – 3q subtelomeric probe; green signal – 3p subtelomeric probe). Note the 3q subtelomeric signals at both ends of the inv dup(3q). C. Whole chromosome 3 paint probe, confirming that the marker consists entirely of chromosome 3 material (arrow). D. G-banded image of the inv dup(3q), with the corresponding ideogram to the right.

This male with wide-spaced nipples, unilateral cryptorchidism, small penis, right clubfoot and left congenital vertical talus had a peripheral blood chromosome analysis performed shortly after birth. Examination of 35 cells demonstrated a normal 46,XY karyotype. He was again evaluated at 14 months of age with findings that also included developmental delay, mild arthrogryposis, skin hyperpigmentation distributed along the lines of Blaschko, a sacral dimple, and an undescended testis on the right. Cranial MRI was normal, but lumbar spine MRI showed cord tethering with hydrosyringomyelia, anterior wedging of the T2 vertebral body, and incomplete closure of the L5 and S1 posterior vertebral elements. A scrotal skin biopsy taken at the time of orchiopexy was submitted for cytogenetic analysis. Eight of 37 cells were found to contain a large SMC. Array CGH using an expanded coverage microarray (see Methods) detected low-level gain of 100 BAC clones from 3q22.3 through 3qter (Fig. 1A). FISH performed on previously G-banded slides using multiple probes from the region of gain confirmed that the marker was derived from the long arm of chromosome 3, and illustrated that the marker had the configuration of an inverted, duplicated 3q (Fig. 1B–D). Also consistent with the array CGH results that the gain did not extend more centromeric than 3q22.3, the marker had no detectable chromosome 3 alpha satellite DNA by FISH (Fig. 1B). Although we cannot absolutely exclude the possibility that the marker contains centromeric material from another chromosome, no other gains were detected by array CGH, and G-banded analysis showed a prominent constriction at one end of the marker at band q26, indicating the likely presence of a neocentromere in that location. The combined results of G-banded analysis, array CGH, and FISH were most consistent with the following configuration of the marker: inv dup(3)(qter→q26→neo→q26→q22.3::q22.3→qter). Parental chromosome analyses were not performed.

### Patient 2

**Figure 2:**
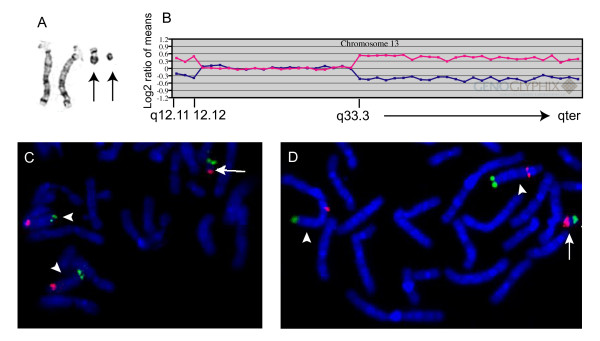
**SMCs from patient 2**. A. G-banded normal chromosome 13 homologues and SMCs (arrows). B. Array CGH plot showing gains of both proximal and distal 13q, with normal copy number in between. C. FISH using BACs RP11-408E5 from 13q12.11 (red signal) and RP11-63L17 from 13q34 (green signal) shows signal from both BACs on the large SMC (arrow), as well as both normal homologues of chromosome 13 (arrowheads). D. FISH using BACs RP11-347L8 from 13q12.13 (red signal) and RP11-63L17 (green) again confirms the presence of both proximal and distal 13q in the large SMC (arrow).

A peripheral blood karyotype was performed on a 2 year-old male with moderate global developmental delay and bilateral anterior segment dysgenesis of the eye in the form of the Axenfeld-Rieger anomaly. In addition, he had macrocephaly, a past history of bilateral inguinal hernias, and several depigmented macules. An MRI was significant for a short, thick corpus callosum, prominent perivascular spaces with thinning of the gray matter, and an enlarged posterior fossa. All 20 cells examined from peripheral blood demonstrated a 48,XY,+mar1,+mar2 karyotype (Fig 2A). The larger marker was approximately one-half the size of a G-group chromosome, and G-banded analysis suggested the presence of satellites. The second marker consisted of a minute fragment. Array CGH using a pericentromeric microarray (see Methods) detected a single-copy gain of 20 BAC clones (approximately 5.3 Mb) from the pericentromeric region of the long arm of chromosome 13 (data not shown). Additional analysis using a targeted microarray (see Methods) revealed not only a gain of the pericentromeric region of chromosome 13, but also single copy gain of approximately 5.5 Mb from the subtelomeric region of chromosome 13 (13q33.3 to q34) (Fig. 2B). The intervening region of chromosome 13 covered by this array (13q14.2 to 13q33.1) did not show copy number alterations (Fig. 2B). FISH using probes from both the proximal (RP11-408E5, 13q12.11 and RP11-347L8, 13q12.13) and distal (RP11-63L17, 13q34) regions of 13q gain confirmed the origin of the larger SMC (Fig. 2C and 2D). Both BACs from proximal 13q hybridized to one arm of the larger SMC, whereas the 13q34 subtelomeric BAC probe hybridized to the opposite arm of the larger SMC. FISH using a probe from the nucleolar organizing region (NOR) hybridized within the middle of the larger SMC, apparently flanked by the 13q proximal long arm segment on one side and by the 13q telomeric segment on the other side (data not shown). Additional FISH using a centromere probe for chromosomes 13 and 21 (D13Z1/D21Z1) showed hybridization only to the larger SMC (data not shown). Thus, none of the probes showed hybridization to the minute SMC, and its origin remains unknown. Based on the combined results of array CGH and FISH, the larger SMC appears to have the following configuration: 13qter→q33.3::p12→q12.12:.

Analysis of 60 peripheral blood metaphase cells from both parents did not show either of the SMCs.

### Patient 3

**Figure 3:**
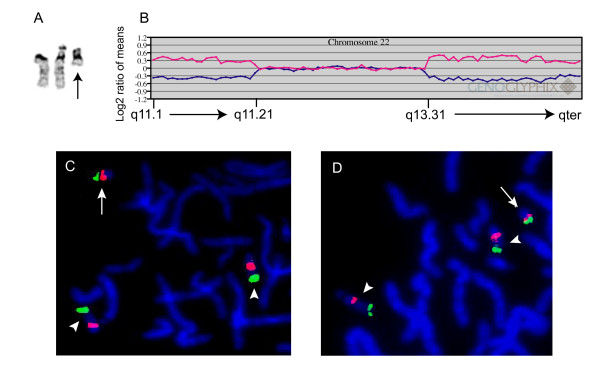
**Chromosome 22 SMC from patient 3**. A. G-banded normal chromosome 22 homologues and SMC (arrow). B. Array CGH plot showing gains of both proximal and distal 22q, but not the intervening region between 22q11.21 and q13.31. C. FISH using BACs RP11-1037C4 from 22q11.1 (red signal) and RP11-676E13 from 22q13.33 (green signal) shows signal from both BACs on the SMC (arrow), as well as both normal homologues on chromosome 22 (arrowheads). D. FISH using BACs CTD-2593O4 from 22q11.21 (red) and RP11-676E13 (green) again confirms the presence of both proximal and distal 22q in the SMC (arrow).

A peripheral blood karyotype from this term newborn female with low birthweight, hydrocephalus, possible partial agenesis of the corpus callosum, preauricular pits, and total anomalous pulmonary venous return showed a small mono-satellited SMC in all 20 metaphase cells examined (Fig. 3A). Array CGH using the pericentromeric microarray detected a single-copy gain of approximately 3.8 Mb of the proximal long arm of chromosome 22 from q11.1 to q11.21 (data not shown), including the VCFS/DiGeorge syndrome critical region (DGS1). Additional analysis using the expanded coverage microarray revealed not only the gain of proximal 22q, but also single-copy gain of approximately 5.2 Mb from 22q13.31 to 22q13.33 (Fig. 3B). The intervening region of chromosome 22, from q11.21 to q13.31, showed no copy number alterations. FISH using BAC clones from both the proximal (RP11-1037C4, 22q11.1 and CTD-2593O4, 22q11.21) and distal (RP11-676E13, 22q13.33) regions of 22q gain confirmed the array findings (Fig. 3C and 3D). Both probes hybridized to the marker in all 60 cells examined. Thus, the most likely configuration of this SMC is: 22pter→q11.21::q13.31→qter. Parental chromosome analyses were not performed.

### Patient 4

**Figure 4:**
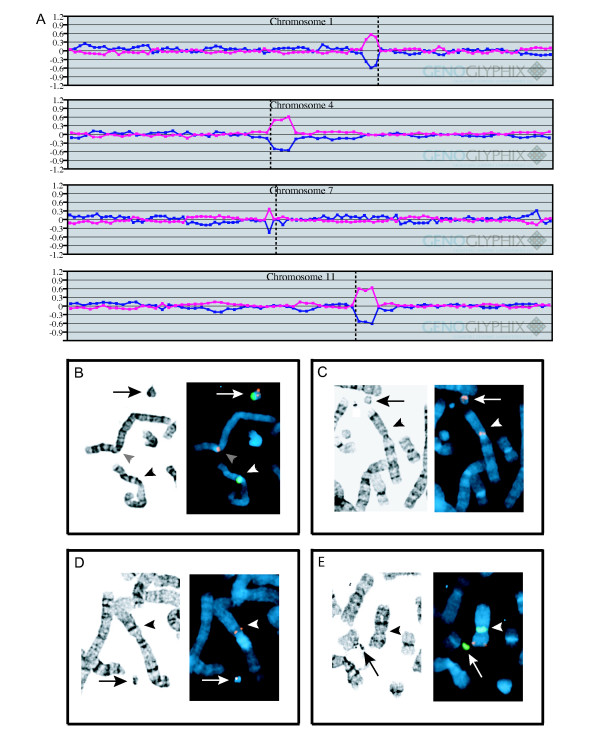
**Characterization of multiple marker chromosomes in patient 4 by array CGH and FISH**. A. Chromosome plots showing pericentromeric gains of 1p, 4q, 7p, and 11q detected by array CGH. B. – D. G-banded and corresponding destained FISH images of the marker chromosomes. B. FISH using BAC RP11-601I15 from 4q12 (orange) and a chromosome 11 alpha satellite probe (green), demonstrating the presence of both chromosome 11 centromere and 4q12 material in the largest SMC (arrow). One normal homologue of chromosome 11 (white arrowhead) and one normal homologue of chromosome 4 (gray arrowhead) are also shown. C. FISH using BAC RP11-1324A7 from 7p11.1, demonstrating signal on a marker chromosome (arrow) and one of the normal chromosome 7 homologues (arrowhead). D. FISH using BAC RP11-527D19 from 1p12, demonstrating signal on a marker chromosome (arrow) and one of the normal chromosome 1 homologues (arrowhead). E. FISH using an X alpha satellite probe (green) and a probe containing the steroid sulfatase (*STS*) gene (orange). The smallest marker chromosome contains X centromere material, but not *STS *(arrow). One normal X chromosome homologue is also shown (arrowhead).

A prenatal ultrasound performed on this female fetus revealed bilateral cleft lip anomaly, ventriculomegaly, and possible agenesis of the corpus callosum. A 48,XX,+2mar/49,XX+3mar karyotype was found at amniocentesis performed at another institution. Interphase FISH to screen for aneuploidies of chromosomes 13, 18, 21, X, and Y showed three X chromosome centromere signals in 40 out of 50 nuclei scored, suggesting that one of the marker chromosomes was derived from the X chromosome. At birth, bilateral cleft lip anomaly, a flattened nasal profile (nasomaxillary hypoplasia), and upslanting palpebral fissures were noted. MRI of the brain showed fusion of the frontal lobes and thalami, as well as partial agenesis of the corpus callosum, consistent with a semilobar holoprosencephaly. No renal anomalies were detected by ultrasound. An echocardiogram showed normal intracardiac structure with a patent ductus arteriosus. A peripheral blood karyotype confirmed the amniocentesis finding of multiple marker chromosomes, but showed four SMCs in the majority of 20 cells examined. A few cells possibly contained five SMCs, although the minute size of some of the markers made them difficult to distinguish from debris. The SMCs ranged in size from minute to one that was approximately one-third the size of a G-group chromosome, and had the appearance of a ring in some cells. Array CGH using the targeted microarray demonstrated gains of the pericentromeric regions of 1p, 4q, 7p, and 11q (Fig. 4A). Numbering of the SMCs from 1 to 4, with SMC 1 representing the largest, and SMC 4 representing the smallest, SMC 1 hybridized with both a chromosome 11 centromere probe (D11Z1) and BAC RP11-601I15 from 4q12 (Fig. 4B), but did not hybridize with a chromosome 4 alpha satellite probe (not shown). SMC 2 hybridized with BAC RP11-1324A7 from 7p11.1 (Fig. 4C), while SMC3 hybridized with BAC RP11-527D19 from 1p12 (Fig. 4D). SMC 4 hybridized with an X chromosome centromere probe (DXZ1) as expected based on the results from amniocentesis. X chromosome alpha satellite sequence is not represented on the targeted microarray that was used for array CGH, and the X chromosome did not show any obvious gains, suggesting that this marker may contain only X centromere material, or a very limited amount of euchromatin. Thus, combined array CGH and FISH results revealed the following configurations of the SMCs in this patient:

SMC1: der(11)r(4;11)(::11q11→11q12.1::4q12::)

SMC2: der(7)(:p11.1:)

SMC3: der(1)(:p12:)

SMC4: der(X)(:p11.1q11.1:)

SKY was also performed by an independent laboratory. Results from five cells, each containing two to three SMCs, showed tentative assignments from chromosomes 1, 5, 7, 11, and 16 (data not shown). FISH did not confirm the presence of chromosome 5 or 16 material in the SMCs. Thus, although SKY classified SMCs derived from chromosomes 1, 7, and 11 material, it did not detect chromosome 4 material in the der(11) SMC, and SMCs in two cells were incorrectly identified as derived from chromosomes 5 and 16. Parental karyotypes were normal.

## Discussion

### Identification of chromosome of origin of SMCs by array CGH

Characterization of the SMCs in this study was performed using either a combination of a pericentromeric microarray and a microarray targeted to specific clinically-relevant regions, or a microarray with expanded coverage. The expanded coverage microarray combines all of the coverage of the first two chips, in addition to having expanded clone coverage within and between known microdeleton/microduplication critical regions. Array CGH was able to identify the chromosome of origin of all but two of the eight SMCs in the four patients who were studied. In one patient (patient 2) who demonstrated two SMCs, array CGH correctly identified the larger of the two markers as a chromosome 13 SMC. FISH using a number of probes from both proximal and distal 13q, including a 13/21 centromere probe, showed hybridization to the larger of the two SMCs only. The second SMC in this patient was minute (see Fig. 2A). In our experience, markers of this size typically fail to show hybridization using 24-color FISH, or yield ambiguous results that are not confirmed with subsequent whole chromosome paints or centromere probes. The X chromosome SMC from patient 4 (see Fig. 4E) that was not identified by array CGH or by SKY was also minute, and may be entirely composed of X centromere material.

### Unexpected structural complexity of SMCs revealed by array CGH

In addition to correctly classifying the chromosomal origin of all but two minute SMCs, array CGH uncovered complex SMC rearrangements that could have been missed if FISH alone had been used for characterization. The chromosome 13 SMC in patient 2 was shown to contain discontinuous segments of proximal and distal 13q by array CGH. FISH using a 13/21 centromere probe, although positive in this SMC, would not have distinguished between these two chromosomes, and it would not have given any information with respect to the regions of chromosome 13 contained within the marker. Twenty-four color FISH might have been able to classify the chromosome of origin of this SMC, but it would also not have revealed the discontinuous nature of the chromosome 13 content. An analogous situation was seen with the chromosome 22 SMC (patient 3) that contains discontinuous segments from 22q. Interestingly, in a recent study of twenty-six patients with nonsatellited SMCs characterized by array CGH, only one was found to have this type of SMC complexity [6]. The analphoid SMC derived from 3q could have been classified using 24-color FISH; however, this technique would not have indicated the region of chromosome 3 involved. A multicolor centromere-specific technique would also not have been able to classify this SMC because of the absence of a conventional chromosome 3 centromere. SKY performed on patient 4, who had multiple SMCs in every cell, resulted in some erroneous chromosome classifications and failed to identify chromosome 4 material in one SMC.

### Utility of FISH for complete SMC characterization

Although array CGH accurately identified the chromosome content of most of the SMCs in the patients presented here, FISH was necessary to determine the structure of the markers. Array CGH correctly defined the segment of 3q comprising the analphoid SMC in patient 1, but FISH ultimately characterized the inverted, duplicated configuration of this marker. By array CGH, the SMC in patient 2 was known to contain discontinuous segments of chromosome 13, but the complex structure of the marker, consisting of short arm material flanked on both sides by different segments of long arm material, was only revealed by FISH. Patients 1 and 4 illustrate the utility of performing FISH on previously G-banded slides. Although array CGH correctly detected extra material from both chromosomes 4 and 11 in patient 4 who had multiple SMCs per cell, FISH after G-banding was necessary to demonstrate that the largest SMC was composed of material from both of these chromosomes. FISH of previously G-banded cells also helped characterize the inv dup(3q) that was present in only approximately 20 percent of metaphase cells, allowing for targeted analysis of cells known to contain the marker.

### SMCs and phenotype

Although the focus of this study was not to perform an extensive phenotype/SMC correlation, array CGH enabled a more accurate correlation.

#### Patient 1: Neocentric chromosome 3 SMC

There are at least eight previously reported SMCs that consist of a neocentric inv dup(3q). Seven of these cases had breakpoints that were substantially more distal (q26, q27, or q28) to the q22.3 breakpoint seen in the patient presented here (reviewed in [7]). The inv dup(3q) was mosaic in all but one case, and had severe clinical effects in all patients. In addition to these seven patients, there was a recent report of a patient with an analphoid, inv dup(3q) and a breakpoint also present in q22.3 [8]. This patient shares some clinical features with the patient presented here and others who have duplications of 3q, including genital abnormalities and various defects in closure of the vertebral column and neural tube. Both patients also exhibited developmental delay and hyperpigmentation along the lines of Blaschko, although the latter finding is not uncommon in patients with chromosomal mosaicism.

#### Patient 2: SMC derived from chromosome 13

For many previously reported acrocentric SMCs that were positive by FISH using a D13Z1/D21Z1 probe, the origin of the SMC from chromosome 13 versus chromosome 21 was not determined, confounding phenotypic correlation with these markers. One of the distinctive findings in patient 2 was that of Axenfeld-Rieger anomaly. Mutations in *PITX2 *(4q25) and *FOXC1 *(6p25), as well as cytogenetic abnormalities involving these loci, have been found in a wide variety of phenotypes that share features with Axenfeld-Rieger anomaly [9,10]. Axenfeld-Rieger anomaly has also been linked to a third locus at 13q14 [9]. This locus may be contained within the SMC found in patient 2, as there is a gap in clone coverage in this region on the targeted array. It is unclear how an additional copy of this locus would cause the phenotype; however, ocular abnormalities, including anterior chamber anomalies, have been described in patients with trisomy 13 [11].

#### Patient 3: SMC derived from chromosome 22

A disproportionate number of SMCs are derived from chromosome 22, and these SMCs vary greatly in structure, 22q content, and phenotypic effect [12]. A subset of chromosome 22 SMCs are associated with cat eye syndrome. These SMCs are bisatellited and contain proximal 22q material, with breakpoints either at the proximal or the distal end of the 22q11 deletion syndrome (DiGeorge/Velocardiofacial syndrome) critical region [13]. The cat eye syndrome phenotype is highly variable and does not correlate with morphology or size of the bisatellited 22q SMC. The chromosome 22 SMC reported here in patient 3 was not bisatellited, and contained not only proximal 22q material including the DiGeorge/VCFS critical region, but also distal 22q, from q13.31 to qter. The phenotypic findings in patient 3 have been described in some patients with trisomy 22/mosaic trisomy 22, although patients with trisomy 22 have multiple additional abnormalities not seen in patient 3. The findings in patient 3 that have been described in trisomy 22 include hydrocephalus, preauricular pits, and heart defects including anomalous venous return [14,15]. Congenital hydrocephalus and hypoplasia of the corpus callosum have also been reported in patients with duplication of 22q13.1 to qter, and 22q13.2 to qter, respectively [16].

#### Patient 4: Multiple SMCs in the same patient

Patient 4 presented with an abnormal phenotype consisting of semilobar holoprosencephaly and bilateral cleft lip, but no other major malformations. This patient was found to have up to four different SMCs in every cell. Whereas a few SMCs have been associated with specific phenotypes, correlation of SMCs with phenotype is problematic due to differences in chromosome of origin, mosaicism, amount of euchromatic material present in the SMC, and the possibility of uniparental disomy of the two normal chromosome homologues. Phenotype/genotype correlations in patients with multiple SMCs may be further complicated by different combinations of SMCs in different cells. Approximately 25–50% of patients with holoprosencephaly have a numerical or structural chromosome abnormality [17]. Trisomy 13 is a frequent numerical abnormality, and structural abnormalities involving almost every chromosome have been reported in these patients [17]. In one study of holoprosencephaly, all of the cytogenetically abnormal patients had malformations in other organ systems in addition to craniofacial malformations, and two-thirds had malformations in three or more organ systems [18]. The absence of additional malformations in this patient with multiple SMCs suggests that these markers do not contain a significant amount of euchromatin.

### Mechanism of formation of SMCs

#### Neocentric chromosome 3 SMC

Although neocentromeres derived from almost all human chromosomes have been reported, hotspots of formation seem to exist [19,20]. The long arm of chromosome 3 is one region that is disproportionately represented amongst neocentromeres [20]. The most common configuration of neocentric chromosomes, including those involving 3q, is that of a supernumerary inverted duplication of the distal arm of a chromosome [7,20]. A frequently proposed mechanism for formation of these markers is that of a U-type exchange between homologous chromosomes during meiosis [21,20]; however, DNA polymorphism studies indicate that neocentric SMCs can form either during meiosis or mitosis [22,23,8].

#### SMCs composed of discontinuous segments of the same chromosome

Two of the markers described in this study fall into this category. We speculated that the most likely configuration of the SMC 13 was: 13qter→q33.3::p12→q12.12. Alternatively, if the marker had the configuration: 13q33.3→qter::p12→q12.12, then this marker may have initially been a ring, and then lost the interstitial segment of 13q, between q12 and q33. It is difficult to distinguish between the two configurations, given the very distal breakpoint in 13q33.

#### Multiple SMCs in the same patient

There are numerous reports of patients with multiple SMCs, particulary multiple ring chromosomes [24,21,25]. Daniel and Malafiej (2003) hypothesized that these additional markers could arise from a superfluous haploid pronucleus in which there is incomplete digestion of some chromosomes, and transfection of these leftover pieces into the zygote [24]. The high proportion of cases with multiple SMCs that are almost always of different centromeric origin is consistent with this hypothesis.

## Conclusion

Based on the different types of SMCs presented here, array CGH is the best initial technique for characterization of these abnormal chromosomes, followed by FISH for complete elucidation of marker structure. We and others have found that array CGH using a chip that not only has good pericentromeric coverage, but also adequate overall genome coverage, is necessary for complete SMC characterization [6,26]. Improved SMC characterization, facilitated by array CGH, will allow for more accurate SMC/phenotype correlation in the future. More accurate genotype/phenotype correlation has also been recently reported for ring chromosomes characterized by array CGH in patients who have a 46 chromosome count [27]. Although array CGH using chips that provide comprehensive genome coverage may become the technology of choice for initial characterization of SMCs, G-banded and FISH analyses are still indispensable for determining the structure and level of mosaicism of these chromosomes. G-banded analysis may also be useful for detecting low-level mosaic SMCs that could potentially be missed by array CGH.

## Methods

### SMCs

SMCs were originally identified by conventional cytogenetic analysis performed in the Children's Hospital and Regional Medical Center Cytogenetics Laboratory. Array CGH was subsequently requested by the referring provider to characterize the markers further. Approval for this study was given by the Seattle Children's Hospital Research Institute Institutional Review Board (application #X-07-047).

### Array CGH

Array CGH on all of the cases was performed using arrays developed and manufactured at Signature Genomic Laboratories, LLC (Spokane, WA). One or more of the following arrays was used in each case: 1) a high-density pericentromeric microarray with coverage of all 43 unique human pericentromeric regions (Signature MarkerChip™ Version 1.0 [28]); 2) a targeted microarray (SignatureChip^® ^Version 4.0 [29,30]; and 3) an expanded coverage microarray (SignatureChip WG™ Version 1.0) [31]. Isolation and labeling of DNA, microarray hybridization, and microarray analysis were performed as described previously [28].

### FISH

All abnormalities detected by array CGH were confirmed and visualized by metaphase or interphase FISH using one or more BAC clones determined to be abnormal by array CGH [32]. In some cases, FISH using commercially available probes (Abbott Molecular Inc., Des Plaines, IL or Cytocell Technologies, Cambridge, U.K.) was also performed with the ThermoBrite denaturation/hybridization system according to the manufacturer's procedure (Abbott Molecular Inc.). For FISH after G-banding, oil was removed by soaking slides in fresh xylene substitute (Thermo Fisher Scientific, Waltham, MA) for 12 minutes. The slides were then placed in methanol for 12 minutes, air-dried, rehydrated in an ethanol series, fixed in 1% formaldehyde/1 × PBS/50 mM MgCl_2 _for 7 minutes, washed in 1 × PBS for 5 minutes, dehydrated in an ethanol series, and air-dried before hybridization using the ThermoBrite system.

### Spectral Karyotyping (SKY)

SKY was performed using Applied Spectral Imaging, Inc. (Vista, CA) SkyPaint^® ^probes according to the manufacturer's protocol. Image acquisition was accomplished using the SpectraCube^® ^spectral imaging system (Applied Spectral Imaging, Inc., Vista, CA) according to the manufacturers' instructions.

## List of abbreviations

Array CGH: microarray comparative genomic hybridization; FISH: fluorescence *in situ *hybridization; SKY: spectral karyotyping; SMC: supernumerary marker chromosome.

## Competing interests

Beth Torchia is a salaried employee of Signature Genomic Laboratories.

## Authors' contributions

KT, KO, and BT directed the cytogenetic studies, interpreted the results, and drafted the manuscript. MH, AH, IG, and MR contributed to the phenotype/genotype correlations and helped revise the manuscript. TN interpreted the SKY results and helped revise the manuscript. All authors read and approved the final manuscript.
